# *E2F1* somatic mutation within miRNA target site impairs gene regulation in colorectal cancer

**DOI:** 10.1371/journal.pone.0181153

**Published:** 2017-07-13

**Authors:** Camila M. Lopes-Ramos, Bruna P. Barros, Fernanda C. Koyama, Paola A. Carpinetti, Julia Pezuk, Nayara T. S. Doimo, Angelita Habr-Gama, Rodrigo O. Perez, Raphael B. Parmigiani

**Affiliations:** 1 Centro de Oncologia Molecular, Hospital Sírio-Libanês, São Paulo, Brazil; 2 Ludwig Institute for Cancer Research, São Paulo, Brazil; 3 Angelita & Joaquim Gama Institute, São Paulo, Brazil; 4 University of São Paulo School of Medicine, São Paulo, Brazil; Universitat de Barcelona, SPAIN

## Abstract

**Background:**

Genetic studies have largely concentrated on the impact of somatic mutations found in coding regions, and have neglected mutations outside of these. However, 3’ untranslated regions (3' UTR) mutations can also disrupt or create miRNA target sites, and trigger oncogene activation or tumor suppressor inactivation.

**Methods:**

We used next-generation sequencing to widely screen for genetic alterations within predicted miRNA target sites of oncogenes associated with colorectal cancer, and evaluated the functional impact of a new somatic mutation. Target sequencing of 47 genes was performed for 29 primary colorectal tumor samples. For 71 independent samples, Sanger methodology was used to screen for *E2F1* mutations in miRNA predicted target sites, and the functional impact of these mutations was evaluated by luciferase reporter assays.

**Results:**

We identified germline and somatic alterations in *E2F1*. Of the 100 samples evaluated, 3 had germline alterations at the MIR205-5p target site, while one had a somatic mutation at MIR136-5p target site. *E2F1* gene expression was similar between normal and tumor tissues bearing the germline alteration; however, expression was increased 4-fold in tumor tissue that harbored a somatic mutation compared to that in normal tissue. Luciferase reporter assays revealed both germline and somatic alterations increased *E2F1* activity relative to wild-type *E2F1*.

**Conclusions:**

We demonstrated that somatic mutation within *E2F1*:MIR136-5p target site impairs miRNA-mediated regulation and leads to increased gene activity. We conclude that somatic mutations that disrupt miRNA target sites have the potential to impact gene regulation, highlighting an important mechanism of oncogene activation.

## Introduction

Genetic studies aimed at understanding tumorigenesis have been primarily focused on detecting mutations in the coding region of genes, but recent work has also highlighted the importance of non-coding regulatory regions. We now know that mutations in the 3’ untranslated regions (3’UTR) have the potential to disrupt or create microRNA (miRNA) target sites, and such sites may mediate an important mechanism of oncogene activation or tumor suppressor inactivation, respectively [1].

More than 60% of human protein coding genes are predicted to contain conserved targets of miRNAs, mostly located within the 3'UTRs [2]. Thus, miRNAs play vital regulatory roles in cell differentiation, proliferation, apoptosis, and other processes that are altered in cancer: miRNAs can function as an oncogene or tumor suppressor depending on the messenger RNA (mRNA) repertoire that they regulate. miRNAs exert their regulatory function by binding to a mRNA target and destabilizing mRNA and/or repressing translation [3]. Genetic alterations at miRNA target sites may significantly modify miRNA-mRNA interactions and thereby impair the mRNA downregulation that is imposed by miRNAs. In fact, germline mutations within miRNA target sites can impact cancer risk and treatment outcomes [4]. For example, a single nucleotide polymorphism (SNP) within a let-7 target site in *KRAS* is associated with increased risk for non-small cell lung carcinoma [5], ovarian cancer [6] and triple negative breast cancer [7]: the variant allele enhances *KRAS* expression *in vitro* by reducing let-7-mediated suppression [5]. An increase in the risk of colorectal cancer (CRC) has also been demonstrated for genetic variants that disrupt miRNA target sites within the *CD86* and *INSR* genes [8].

Based on the functional impact of germline alterations within miRNA target sites, identification of somatic mutations distinguishing cancer from normal cells is imperative for more fully understanding a major mechanism of tumorigenesis. The first somatic mutation affecting gene expression by generation of a new miRNA target site was reported for acute myeloid leukemia [9]. TNF alpha induced protein 2 (*TNFAIP2*) is a known target for transcriptional repression by the *PML-RAR* oncogene, and as such a *TNFAIP2* somatic mutation was found to repress translation in this gene in a miRNA-dependent fashion. Nevertheless, the miRNA that mediates this effect has not been identified. Recently, as a consequence of extensive data provided by whole genome sequencing of cancer cells, the SomamiR database has integrated miRNA-related mutations in cancer [10], and has permitted wide identification of somatic mutations that create or disrupt miRNA target sites. The functional impact of most of these mutations has not been investigated, though, we presume that mutations disrupting miRNA target site could confer oncogenic properties to genes such as E2F transcription factor 1 (*E2F1)*.

*E2F1* is a transcription factor with a key role in regulating cell cycle, differentiation and oncogenesis; it is tightly regulated by the retinoblastoma tumor suppressor gene, and exerts its functions through regulation of genes that are required for chromosomal DNA replication and cell cycle progression [11–13]. Amplification and/or deregulated expression of the *E2F1* gene have been described in several cancer types, including breast, lung and CRC, and high levels of *E2F1* are frequently correlated with high-grade tumors or metastases and poor prognosis [13–17].

In this study, we have systematically searched for somatic mutations within predicted miRNA target sites of several important oncogenes in CRC, and have evaluated the functional impact of a new *E2F1* somatic mutation. We show that the *E2F1* mutation within the MIR136-5p target site increases gene expression caused by the loss of miRNA regulation.

## Materials and methods

**Fig 1:**
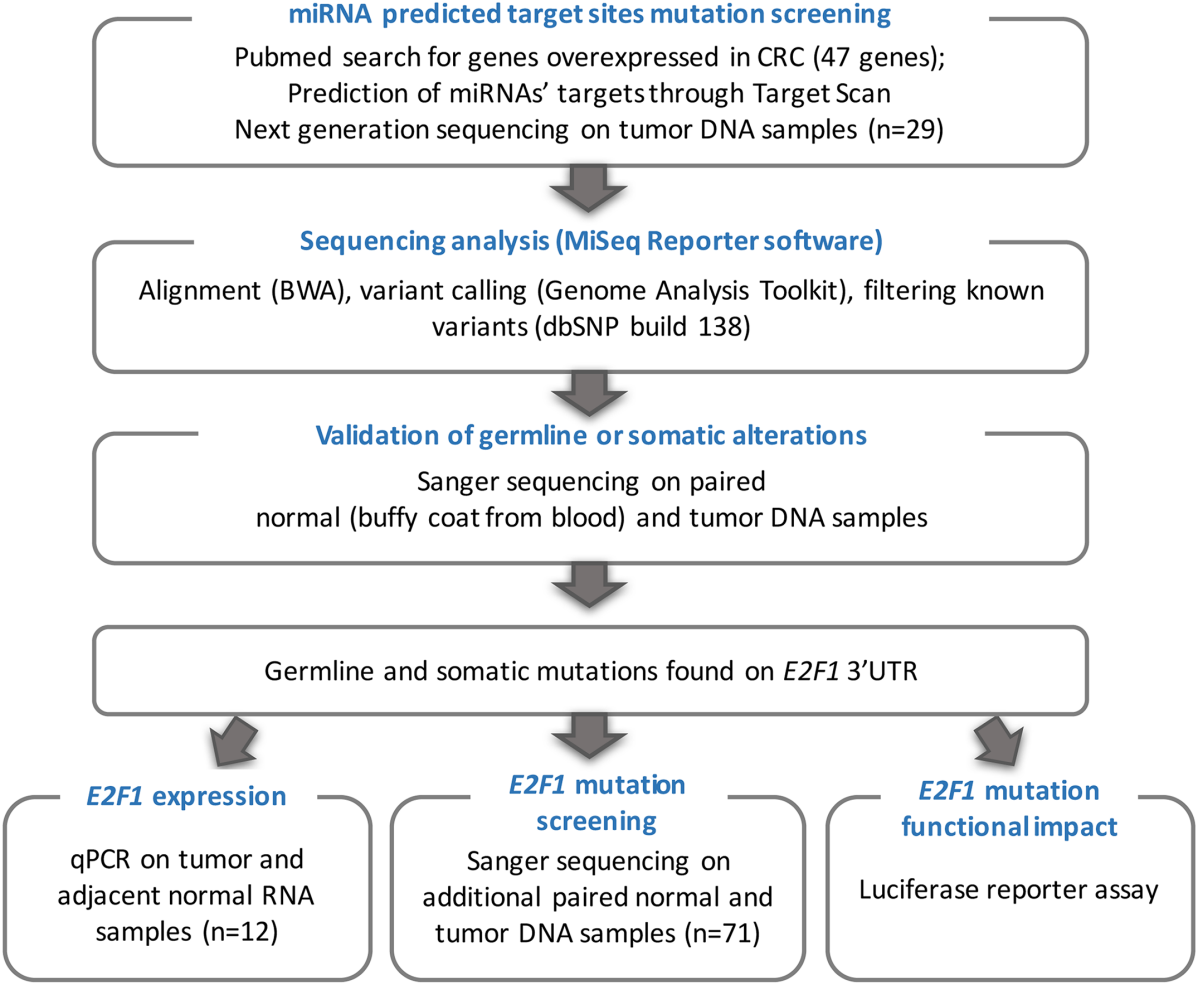
Study overview. BWA: Burrows-Wheeler Aligner.

Our study strategy is illustrated in Fig 1.

### Clinical samples

This study was approved by Hospital Sírio-Libanês (São Paulo, Brazil) ethics committee (AVAP:RBP84), and all study participants signed an informed consent form prior to sample collection. A total of 100 patients with histologically confirmed diagnoses of colorectal adenocarcinoma stages II-IV (according to the Union for International Cancer Control—UICC) were included in this study. Primary tumor samples were collected during surgery, snap frozen in liquid nitrogen, and stored at -80°C. Only tumor fragments with ≥80% of cancer cells were used. Peripheral blood was also collected from each patient, and the buffy coat fraction obtained. All biospecimens used were part of Hospital Alemão Oswaldo Cruz biobank (São Paulo, Brazil).

### miRNA target sites capture and sequencing

Genomic DNA was obtained with use of a standard phenol-chloroform extraction method. Target sequencing was performed for 47 genes that are frequently overexpressed in CRC (see Table 1). The targeting was designed with use of SureDesign software (Agilent Technologies Inc.), and covered all conserved mammalian miRNA target sites for conserved miRNA families, as predicted by TargetScan Human 5.1 [18]. The total region sequenced included 14.1 kb bases and had a mean sequencing depth of 180x (varying from 120x to 290x). Target enrichment and library preparation were accomplished using the HaloPlex target enrichment system (Agilent Technologies Inc.). Samples were DNA-barcoded and a pool of 29 samples was sequenced in a single run on an Illumina MiSeq platform with 2×250 bp read length.

**Table 1:** List of genes screened by next-generation sequencing for genetic alterations at miRNA predicted target sites.

Official Symbol	Official Full Name	Gene ID	Official Symbol	Official Full Name	Gene ID
*BCL2*	BCL2, apoptosis regulator	596	*MAP3K3*	mitogen-activated protein kinase kinase kinase 3	4215
*DHFR*	dihydrofolate reductase	1719	*MAP3K4*	mitogen-activated protein kinase kinase kinase 4	4216
*DICER1*	dicer 1, ribonuclease III	23405	*MAP3K5*	mitogen-activated protein kinase kinase kinase 5	4217
*DNMT3A*	DNA methyltransferase 3 alpha	1788	*MAP3K7*	mitogen-activated protein kinase kinase kinase 7	6885
*DTL*	denticleless E3 ubiquitin protein ligase homolog	51514	*TAB1*	TGF-beta activated kinase 1 (MAP3K7) binding protein 1	10454
*E2F1*	E2F transcription factor 1	1869	TAB2	TGF-beta activated kinase 1 (MAP3K7) binding protein 2	23118
*E2F2*	E2F transcription factor 2	1870	*TAB3*	TGF-beta activated kinase 1 (MAP3K7) binding protein 3	257397
*E2F3*	E2F transcription factor 3	1871	*MAP3K9*	mitogen-activated protein kinase kinase kinase 9	4293
*EGFR*	epidermal growth factor receptor	1956	*MAP4K3*	mitogen-activated protein kinase kinase kinase kinase 3	8491
*ERBB3*	erb-b2 receptor tyrosine kinase 3	2065	*MAP4K4*	mitogen-activated protein kinase kinase kinase kinase 4	9448
*EVI1*	ecotropic viral integration site 1	733318	*MAP7D1*	MAP7 domain containing 1	55700
*EVI5*	ecotropic viral integration site 5	7813	*MAPK1*	mitogen-activated protein kinase 1	5594
*HIF1A*	hypoxia inducible factor 1 alpha subunit	3091	*MAPK6*	mitogen-activated protein kinase 6	5597
*HSPA5*	heat shock protein family A (Hsp70) member 5	3309	*MAPK7*	mitogen-activated protein kinase 7	5598
*IGF1*	insulin like growth factor 1	3479	*PIK3C2A*	phosphatidylinositol-4-phosphate 3-kinase catalytic subunit type 2 alpha	5286
*IGF1R*	insulin like growth factor 1 receptor	3480			
*IGF2R*	insulin like growth factor 2 receptor	3482	*PIK3IP1*	phosphoinositide-3-kinase interacting protein 1	113791
*IRS1*	insulin receptor substrate 1	3667	*PIK3R1*	phosphoinositide-3-kinase regulatory subunit 1	5295
*ITGA6*	integrin subunit alpha 6	3655	*SIRT1*	sirtuin 1	23411
*KLF2*	Kruppel like factor 2	10365	*SOX2*	SRY-box 2	6657
*KLF5*	Kruppel like factor 5	688	*SOX4*	SRY-box 4	6659
*KRAS*	KRAS proto-oncogene, GTPase	3845	*VEGFA*	vascular endothelial growth factor A	7422
*MAP2K1*	mitogen-activated protein kinase kinase 1	5604	*YES1*	YES proto-oncogene 1, Src family tyrosine kinase	7525
*MAP3K1*	mitogen-activated protein kinase kinase kinase 1	4214	*YY1*	YY1 transcription factor	7528

### Sequencing analysis

Sequencing analysis was performed using MiSeq Reporter software. Sequencing reads were aligned against the human reference genome (hg19; GRCh 37) by the Burrows-Wheeler Aligner (BWA). Given that this was a paired-end run, we eliminated paired reads if either one of the reads did not align to the reference, or aligned to different chromosomes. Aligned sequencing data have been deposited in the National Center for Biotechnology Information (NCBI) Sequence Read Archive (SRA) with the accession number of SRP108027, and with the BioProject accession number of PRJNA386377. The Genome Analysis Toolkit was used to call variants: criteria consisted of at least 10x coverage, variation frequency of >20%, and base quality of >30. Since we were interested in somatic variants, we next filtered the identified variants that had been annotated in the Single Nucleotide Polymorphisms database (dbSNP) build 138 (common genetic variants with minor allele frequency greater than or equal to 1%).

### Capillary sequencing validation

Identified variants were validated using the automated DNA Sanger sequencing ABI3130XL (Applied Biosystems). Besides the tumor sample, a matched sample from buffy coat was also sequenced, to evaluate if the identified variant comprised exclusively tumor tissue. The following primers were used for Polymerase Chain Reaction (PCR): YES proto-oncogene 1, Src family tyrosine kinase (*YES1*) *YES1*_Fw: TGGGTGACAGCATGGTAATG, *YES1*_Rv: TTTCCCCTTTGATTGGACAG, *E2F1*_Fw: AATCAAATCGGGCACGGAC, *E2F1*_Rv: GGTGTGTATGTGCATGCAGC (Fw: forward; Rv: reverse). PCR products were sequenced using the same forward or reverse primers, along with the BigDye Terminator v3.1 Cycle Sequencing Kit (Applied Biosystems), and sequences were analyzed with FinchTV (Geospiza).

### Gene expression analysis

Total RNA was extracted from tumor, or from adjacent normal tissue, with Trizol reagent (Invitrogen); RNA quality was evaluated using 2100 Bioanalyzer (Agilent). RNA was treated with DNase (Turbo DNA Free, Ambion), reverse-transcribed to cDNA (Superscript III, Invitrogen), and quantified by real time quantitative PCR (qPCR) (SYBR Green PCR Master Mix, Thermo Fisher Scientific) using the 7900HT Fast System (Applied Biosystems). *E2F1* gene expression was evaluated with use of the following primers, Fw: CTGCCCATCCGGGACAACA, Rv: CCATCATCTCCCCCCTCATCC. Pumilio RNA binding family member 1 (*PUM1*) and hydroxymethylbilane synthase (*HMBS*) genes were used for normalization (*PUM1*_Fw: TGTACTTACGAAGAGTTGCGATGTG, *PUM1*_Rv: CCAGGCCAGCGGAAGAT, *HMBS*_Fw: GGCAATGCGGCTGCAA, *HMBS*_Rv: GGGTACCCACGCGAATCAC).

A Qiagen miScript PCR system was used for miRNA expression analysis: cDNA was generated with miScript II RT kit (Qiagen) and 6 ng used as template in each qPCR reaction (miScript SYBR Green PCR kit, Qiagen). RNA, U6 small nuclear 1 (*RNU6b*) expression was used for normalization.

The expression levels of *E2F1*, MIR205-5p, and MIR136-5p were analyzed for 12 patients (total number of patients used for DNA target sequencing that had total RNA available for tumor and normal adjacent tissues in the tumor tissue biobank). For each patient, the tumor/normal relative expression was calculated, based on the ΔΔCT method [19], with the respective normal sample serving as a reference. qPCR reactions were performed in triplicate, and the standard error of the mean (SEM) reported.

### Luciferase reporter assay

Part of the *E2F1* 3'UTR (867bp) was amplified from the tumor sample of the two patients who harbored the mutations found. PCR was performed with the following primers: Fw: ATTCTCGAGGCTAGGAGGCTGAGCAAGC, Rv: CCTCTAGAACCAAAGCAGGAGGGAACAG. PCR products were digested with XhoI and XbaI, and cloned into the XhoI and XbaI sites of the pmirGLO Dual-Luciferase miRNA Target Expression Vector (Promega). Clone sequences were verified to retrieve at least one clone with the *E2F1* wild-type (WT) sequence, one mutated at the MIR205-5p target site, and one mutated at MIR136-5p target site.

The human colorectal tumor cell line HCT116 was obtained from American Type Culture Collection (ATCC), and cells were cultured following manufacturer’s recommendations. Cell line authentication test was performed using GenePrint (Promega). For stable transfection, luciferase constructs were transfected with lipofectamine 3000 reagent (Life Technologies), and G418-resistant populations were selected.

For reporter expression assays, we selected cells that stably expressed either WT or mutant *E2F1* luciferase constructs, and seeded them in 96-well plates (15,000 cells per well). After 48h, cells were transiently transfected with 1pmol mirVana miRNA Mimics (MIR205-5p, MIR136-5p or mimic control) using RNAiMAX Transfection Reagent (Life Technologies), as per manufacturer’s protocol. Cells were incubated for 48h, collected, and luciferase activity was measured using the Dual-Luciferase Reporter Assay (Promega). The luminescent signal was quantified using Infinite 200 PRO (Tecan Group Ltd).

The experimentally validated miRNA:target interactions were reported following the recommended minimal standards in Piletič *et al*. [20]. For miRNA nomenclature, we followed the guidelines provided in Desvignes *et al*. [21].

## Results

### Genetic alteration at miRNA target sites

We investigated somatic mutations located at predicted miRNA target sites of 47 genes frequently overexpressed in CRC based on a literature search (Table 1). Table 2 describes clinical and demographics data of patients included in the study. An initial screen by next-generation sequencing (NGS) was done for 29 CRC samples, and we found 3 single nucleotide variants in three different patients. Two samples had alterations on *E2F1* gene (at MIR136-5p or MIR205-5p target sites), and one sample had an alteration on *YES1* gene (at the MIR504-5p target site) (Table 3). Validation was performed through Sanger sequencing methodology, and every alteration was confirmed in the same tumor sample. We also sequenced normal tissue from the buffy coat of the same patients. *YES1*:MIR504-5p and *E2F1*:MIR205-5p alterations were also present in normal tissues, and were thus considered to be germline alterations; nevertheless, the *E2F1*:MIR136-5p alteration was exclusively present in tumor tissue and confirmed as a somatic mutation.

**Table 2:** Clinical and demographics data of patients included in the study.

Patients’ Demographics	
Number of patients	100
Mean age (years ± SD)	62.6 (±12.9)
Gender (n)	
Female	46
Male	54
Tumor location (n)	
Rectum	49
Left colon	25
Transverse colon	7
Right colon	19

**Table 3:** Position and sequencing characteristics of mutations found in miRNA predicted target sites.

Sample	Gene	Interacting miRNA	Chr	Position	Reference	Alteration	Quality	Variant frequency	Sequencing Depth	Type
2	*E2F1*	MIR136-5p	20	32263982	C	T	100	0.24	75	somatic
11	*E2F1*	MIR205-5p	20	32264259	G	A	100	0.29	237	germline
12	*YES1*	MIR504-5p	18	724214	C	T	100	0.53	45	germline

### *E2F1* expression analysis

**Fig 2:**
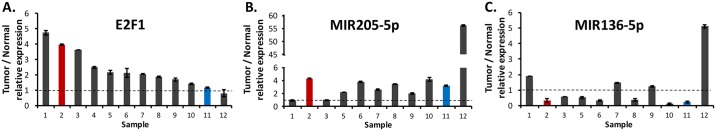
*E2F1* and miRNA expression in a set of patient samples. A) *E2F1* expression in tumor relative to normal tissue was evaluated by qPCR; *PUM1* and *HMBS* expression were used for normalization. B) MIR205-5p and C) MIR136-5p tumor expression relative to normal expression was evaluated by qPCR; *RNU6b* expression was used for normalization. Red: patient #2 carries a somatic mutation at *E2F1*:MIR136-5p target site. Blue: patient #11 carries a germline alteration at *E2F1*:MIR205-5p target site. Dashed line represents the same expression value between normal and tumor tissues. Error bars indicate the SEM of experiments in triplicate.

Given that a mutation at a miRNA target site may impair gene expression, we evaluated *E2F1* gene expression by comparing tumor and normal adjacent tissues. The set of patients used for DNA target sequencing that had total RNA available for tumor and normal adjacent tissues in the tumor tissue biobank were included in this analysis, totalizing 12 patients, including those that were carrying the somatic or germline alterations. Consistent with the important oncogenic role of *E2F1*, 7 patients (58%) had at least 2-fold higher expression of *E2F1* on tumor tissue compared to paired normal tissue (Fig 2A). For patient #11, with *E2F1* germline alteration at the MIR205-5p target site, gene expression in the tumor tissue was similar to that seen in normal tissue. However, *E2F1* gene expression was 4-fold greater on tumor compared to normal tissue of patient #2, who presented a somatic mutation at the MIR136-5p target site.

The functional impact of a mutation present at a miRNA target site depends primarily on miRNA expression. We thus confirmed MIR205-5p and MIR136-5p expression in tumor tissue: MIR205-5p expression was elevated in tumor compared to normal tissue (Fig 2B); and MIR136-5p expression was reduced in tumor compared to normal tissue for most of analyzed samples (Fig 2C).

### Mutation functional impact

Some nucleotide positions are crucial for efficient binding of miRNA to mRNA, and its repression function. For instance, pairing to the seed region is often sufficient for the specificity of functional binding [22], and the *E2F1*:MIR136-5p somatic mutation is located exactly at the position where the MIR136-5p seed region pairs. Additional pairing outside the seed region might also be important for miRNA functional binding, specifically for *E2F1*:MIR205-5p, the identified germline alteration leads to complementarity loss between the mRNA target and miRNA 15^th^ position.

We tested whether the identified mutations had a functional impact on *E2F1* expression. HCT116 cell line that stably expressed WT or mutant *E2F1* 3'UTR (cloned downstream of luciferase reporter gene) were transiently transfected with miRNA mimics. First, we experimentally validated that MIR205-5p and MIR136-5p could, in fact, negatively regulate *E2F1* expression, confirming TargetScan predicted target sites. We experimentally validated these miRNA:target interactions performing luciferase reporter assays on the colorectal tumor cell line HCT116. More specifically, we validated the interaction between *Homo sapiens* (9606) *MIR205* (406988) and *E2F1* (1869) within the 3’UTR genomic location hg19 chr20:32263292–32264537, and the mature miRNA seed region at chr20:32264247–32264253. We also validated the interaction between *Homo sapiens* (9606) *MIR136* (406927) and *E2F1* (1869) with mature miRNA seed region at chr20:32263982–32263988.

**Fig 3:**
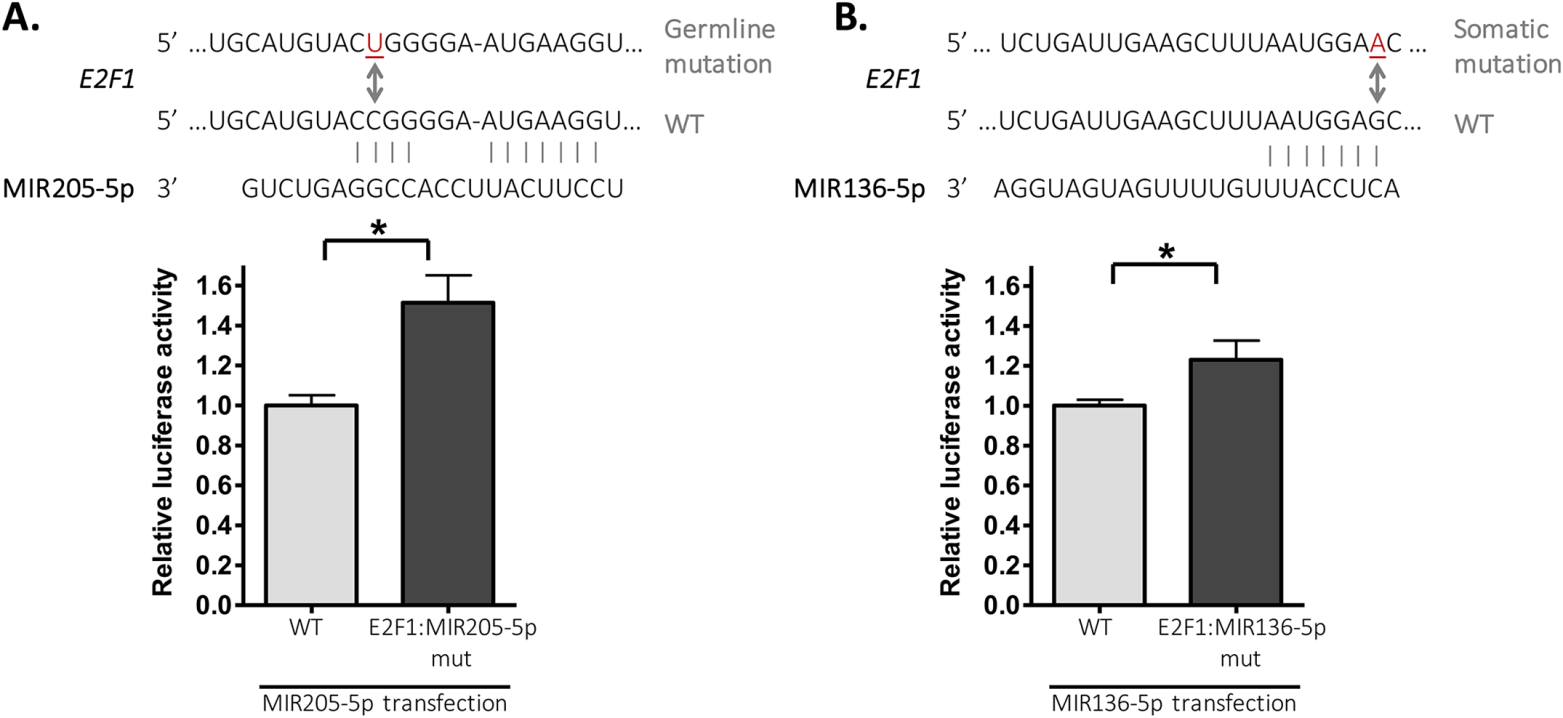
Reporter assays demonstrate that constructs containing *E2F1* mutations enhance gene activity relative to the wild-type (WT) construct. Scheme shows pairing between miRNA and *E2F1* WT or mutated sequence, as predicted by TargetScan. HCT116 cells were manipulated to stably express luciferase reporter constructs that contain the partial sequence of *E2F1* 3'UTR WT, mutated at the MIR205-5p target site (A) or at the MIR136-5p target site (B). Cells were transiently transfected with control miRNA or miRNA mimics (MIR205-5p (A) or MIR136-5p (B)); luciferase activity was assayed after 48h. Values show luciferase activity relative to WT after normalizing to the respective miRNA control-transfected cells, and indicate the average of three independent experiments ± standard deviation (SD). *P≤0.02, unpaired t-test.

Next, we showed that, upon MIR205-5p overexpression, there was a statistically significant 50% higher luciferase activity from the construct containing the *E2F1*:MIR205-5p mutation, relative to the *E2F1* WT construct (Fig 3A). Additionally, we demonstrated a 20% higher luciferase activity from the construct that contained the *E2F1*:MIR136-5p mutation, after MIR136-5p overexpression (Fig 3B).

Overall, these results indicate that germline and somatic *E2F1* mutations, located at the MIR205-5p and MIR136-5p target sites, respectively, can impair miRNA-mediated regulation and lead to increased gene activity compared to WT *E2F1*.

### *E2F1* mutation screening

To better evaluate the incidence of the *E2F1* mutation, 71 independent CRC samples were screened for *E2F1* mutations through Sanger sequencing. All predicted miRNA binding sites within the 3'UTR of *E2F1* were evaluated, which consisted of 6 conserved mammalian miRNA target sites for conserved miRNA families, as predicted by TargetScanHuman 5.1. We detected 2 germline alterations at MIR205-5p target site (S1 Table). Notably, the same germline alteration previously found through NGS at the MIR205-5p target site was also found in these 2 additional samples. This alteration is not part of the Common SNPs (138) (the subset of dbSNP used during our initial screening to filter out germline alterations, which includes only SNPs with ≥ 1% minor allele frequency); however, it has already been reported in dbSNP 138 as a low frequency allele (rs149816386) and its frequency in the 1000 Genomes Project is 0.872% [23].

In brief, of the 100 samples evaluated for mutations on *E2F1* miRNA target sites, 3 samples had germline alterations at the MIR205-5p target site, and one sample had a somatic mutation at the MIR136-5p target site. Clinical and demographics data of these patients are similar to those patients with no mutations identified (S2 Table).

## Discussion

Somatic mutations within gene regulatory elements may contribute to tumorigenesis by affecting gene expression. miRNA target sites are among the most important post-transcriptional regulatory elements, and somatic mutations on such elements may result on oncogene activation or tumor suppressor gene inactivation. We have applied NGS technology to undertake a wide screen for genetic alterations in predicted miRNA target sites of important oncogenes in CRC.

Of the 47 genes we sequenced, alterations were found in 2 (*E2F1* and *YES1*). A germline alteration was found in *YES1*, and germline as well as somatic alterations were found in *E2F1*. Because most of the analyzed genes did not harbor this type of mutation, we conclude that somatic mutations at miRNA target sites occur at a low frequency in CRC. Note, however, that our sample size was limited. Additional 71 CRC samples were screened for *E2F1*, and we identified 2 new samples with germline alterations located at the MIR205-5p target site. *E2F1* somatic mutation was identified in one sample out of the total 100 analyzed, indicating this is likely a rare event.

Emerging evidence indicates that germline alterations within miRNA target sites alter variant allele expression, and may contribute to cancer susceptibility [4,24]. Our study used reporter assays and showed that after MIR205-5p overexpression, mutated *E2F1* (at the miRNA target site) has a 50% higher luciferase activity when compared to WT *E2F1*. Regulation of *E2F1* by *MIR205* has already been validated for melanoma, where overexpression of *MIR205* directly inhibits *E2F1* expression and prevents tumor progression *in vivo* [25]. Accordingly, *MIR205* is frequently downregulated in melanoma [25]. Thus, the functional impact of *E2F1*:MIR205-5p germline alteration observed in our study represents a different mechanism of *E2F1* overexpression, one that is not dependent on the deregulation of miRNA expression. Nevertheless, a larger set of samples from patients with cancer or healthy should be evaluated to verify a direct association of this germline alteration with CRC susceptibility.

In contrast to the established impact of germline alterations within miRNA target sites, somatic mutations are still relatively underexplored. Using the extensive data from cancer genome sequencing projects, SomamiR database has catalogued more than 27,000 somatic mutations that alter predicted or experimentally identified miRNA target sites, showing the potential impact that these events might have on tumorigenesis [10]. Nonetheless, to date only one study has demonstrated the functional impact of a somatic mutation that creates a miRNA target site within *TNFAIP2*, in acute myeloid leukemia [9]. In particular, 10 somatic mutations for *E2F1* have been described by SomamiR. They were identified in different cancer types, including CRC, and predicted to alter the target sites for *MIR4321*, *MIR4662a*, *MIR181b*, and *MIR10a*. The authors of these studies were not directly searching for miRNA target site mutations, but the frequency of these findings support the significance of describing the functional impact of a new somatic mutation in *E2F1*.

*E2F1* is an important oncogene frequently correlated with high-grade tumors or metastases and poor prognosis [13]. Overexpression of the *E2F1* and its impact on cancer progression have recently been associated with miRNA deregulation in different types of cancer: melanoma, lung cancer, glioma and CRC, where marked reduction in expression was observed for *MIR205*, *MIR493*, *MIR329* and *MIR362*, respectively [25–28]. Additionally, restoring expression for these miRNAs *in vitro* inhibited cell proliferation, by directly downregulating *E2F1*. We report a new somatic mutation at *E2F1*, and document that *E2F1* mRNA expression is 4-fold higher in tumor compared to the normal tissue. Since the MIR136-5p expression itself was downregulated in the tumor tissue, we could not rule out whether *E2F1* higher expression was only due to the target site mutation. Still, our *in vitro* reporter assays confirmed that this somatic mutation indeed impairs MIR136-5p regulation of *E2F1*.

## Conclusions

Previous studies have demonstrated a tight association between downregulation of specific miRNAs and *E2F1* overexpression in tumor tissues. Using a different approach, we looked for somatic mutations in miRNA predicted target sites located on *E2F1* 3’UTR in CRC samples. Thus, we found that somatic mutations at miRNA target sites can impair miRNA regulation in CRC and lead to an increase of *E2F1* expression relative to normal tissue, which in turn could have oncogenic properties. Considering that different molecular mechanisms can contribute to *E2F1* overexpression in cancer, further studies will be needed to confirm that *E2F1* somatic mutations within miRNA target site contribute in fact to tumorigenesis.

## Supporting information

S1 Table Variants found in miRNA target sites after *E2F1* mutation screening through Sanger sequencing in 71 independent samples. (PDF)

S2 Table Clinical and demographics data of patients with mutations found in *E2F1* miRNA target sites. (PDF)
