# Supplementary material for: E2F1 somatic mutation within miRNA target site impairs gene regulation in colorectal cancer
Source: PLoS One. 2017 Jul 13;12(7):e0181153. doi: 10.1371/journal.pone.0181153 (PMC5509303; doi:10.1371/journal.pone.0181153)
Supplement: S1 Table — (PDF) [file pone.0181153.s001.pdf]

Table S1. Variants found in miRNA target sites after *E2F1* mutation screening through Sanger sequencing in 71 independent samples.

Sample	Gene	Interacting miRNA	Chr	Position	Reference	Alteration	dbSNP	Type
32	*E2F1*	MIR205-5p	20	32264259	G	A	rs149816386	germline
58	*E2F1*	MIR205-5p	20	32264259	G	A	rs149816386	germline
