# Supplementary material for: E2F1 somatic mutation within miRNA target site impairs gene regulation in colorectal cancer
Source: PLoS One. 2017 Jul 13;12(7):e0181153. doi: 10.1371/journal.pone.0181153 (PMC5509303; doi:10.1371/journal.pone.0181153)
Supplement: S2 Table — (PDF) [file pone.0181153.s002.pdf]

Table S2. Clinical and demographics data of patients with mutations found in *E2F1* miRNA target sites.

Sample	Age	Gender	Tumor location	Staging
2	36	F	Rectum	cT2N0M0
11	65	M	Rectum	pT3N1M1
32	85	F	Left Colon	pT3N0M1
58	77	F	Rectum	pT3N2M0
